# Neighbourhood immigrant density and COVID-19 infection and hospitalisation among healthcare workers in Sweden: a register-based observational study

**DOI:** 10.1136/bmjph-2024-001501

**Published:** 2025-02-26

**Authors:** Chioma Nwaru, Carl Bonander, Huiqi Li, Ailiana Santosa, Jesper Löve, Fredrik Nyberg

**Affiliations:** 1 School of Public Health and Community Medicine, Institute of Medicine, Sahlgrenska Academy, University of Gothenburg, Gothenburg, Sweden; 2 Population Health Sciences Institute, Faculty of Medical Sciences, Newcastle University, Newcastle upon Tyne, UK

**Keywords:** COVID-19, SARS-CoV-2, Epidemiology

## Abstract

**Introduction:**

We investigated whether living in immigrant-dominated neighbourhoods constituted a risk factor for COVID-19 infection and hospitalisation among healthcare workers (HCWs) in Sweden, and if so, whether such exposure exacerbated the risk of COVID-19 among immigrant HCWs.

**Methods:**

We used population-based register data from HCWs aged 20–62 years (N=86 187) resident in 14 Swedish municipalities (3 of which are Sweden’s largest metropolitan cities) on 1 January 2020. Residential neighbourhoods of the HCWs were categorised into three groups: Swedish-dominated, mixed and immigrant-dominated. Multilevel mixed-effects survival regression was used for the association analyses, with control for relevant confounding variables. The results are reported as HRs, with 95% CIs.

**Results:**

From 1 January 2020 to 30 September 2022, we recorded 39 746 COVID-19 infections and 860 COVID-19-related hospitalisations. Except during the first wave of the pandemic, living in immigrant-dominated (adjusted HR 0.98; 95% CI 0.94 to 1.01) or mixed (adjusted HR 1.02; 95% CI 0.99 to 1.05) neighbourhoods was not associated with COVID-19 infection, but living in these areas was associated with an increased risk of having COVID-19-related hospitalisation throughout the study period. Immigrant HCWs, regardless of their neighbourhood of residence, had approximately 2-fold higher risk of being hospitalised for COVID-19 than non-immigrant HCWs living in Swedish-dominated neighbourhoods.

**Conclusions:**

Among HCWs in Sweden, neighbourhood immigrant density constituted a risk factor for COVID-19-related hospitalisation. However, immigrant HCWs had an elevated risk of COVID-19-related hospitalisation regardless of where they lived.

**WHAT IS ALREADY KNOWN ON THIS TOPIC**

- There are disparities in COVID-19 infection and hospitalisation among healthcare workers (HCWs), but the contribution of residential neighbourhood immigrant densities to the disparities has not yet been explored.

**WHAT THIS STUDY ADDS**

- HCW living in immigrant-dominated neighbourhoods had an elevated risk of COVID-19 infection but only during the first wave of the pandemic.
- During the entire study period, living in immigrant-dominated neighbourhoods was associated with an increased risk of having COVID-19-related hospitalisation.
- An elevated risk of COVID-19-related hospitalisation was observed among immigrant HCWs irrespective of the neighbourhood they lived in.

**HOW THIS STUDY MIGHT AFFECT RESEARCH, PRACTICE OR POLICY**

- The persistent increased risk of COVID-19-related hospitalisation among immigrant HCWs is a cause for concern. Further research is needed to identify the root causes of this excess risk.

## Introduction

 Healthcare workers (HCWs) were among those who championed the fight against the highly infectious COVID-19 disease, which began in Wuhan, China in 2019 and quickly spread to become a global crisis in early 2020. Throughout the pandemic, HCWs worked on-site, putting their own life and that of their loved ones at risk. Studies have consistently found a higher risk of COVID-19 infection among HCWs than non-HCWs,^1 2^ but the mechanisms underlying the disparity are not yet fully known.^3^ Attempts have been made to understand the role of occupational exposure, but the findings show inconsistent results. Some suggest that working in specific healthcare roles (eg, nurses) and work environments (eg, COVID-19 wards) are important risk factors,^3^,^5^ while others show conflicting patterns.^6 7^

Studies (from Western countries) also suggest that HCWs who are immigrants or belong to ethnic minorities are at even greater risk of COVID-19 infection than their peers.^1 8 9^ This finding persists even after controlling for work-related factors. For example, in several UK-based studies,^5 10 11^ black and Asian HCWs were found to have an elevated risk of COVID-19 infection after controlling for age, sex, socioeconomic status, occupational role and work location. In a previous longitudinal study of immigrant HCWs in Sweden,^12^ we found that, despite being exposed to similar jobs, African-born and Asian-born physicians had a higher risk of COVID-19 infection than European-born physicians. Given that work-related factors (job role, work location) do not completely explain the differences in COVID-19 outcomes between HCWs and non-HCWs, as well as among HCWs, additional research is needed to identify other factors that may underlie the disparities.

Neighbourhood immigrant density is a well-known determinant of health. It has been linked to increased risk of cardiovascular^13^ and infectious diseases (eg, tuberculosis)^14^ on the one hand, and decreased risk of mental health problems on the other,^15^ indicating that it can act either as barriers or facilitators of health-related lifestyle and stress that ultimately impact health outcomes.^16^ With regard to COVID-19, neighbourhoods with high concentration of immigrants, which are frequently characterised by poverty, poor housing conditions, limited access to health services and other forms of socioeconomic deprivation, can exacerbate the spread of the SARS-CoV-2 virus and make disease control measures (eg, isolation) difficult to implement.^9 14 17^ Studies from the USA,^18^,^21^ Canada^22^ and Sweden,^23^ where disproportionate risk of COVID-19 cases and deaths were reported in areas dominated by immigrants and racial and ethnic minorities, provide support for this notion. Nonetheless, studies have yet to investigate and quantify the role of neighbourhood immigrant density in COVID-19 disparities among HCWs.

Here, we investigated whether living in immigrant-dominated neighbourhoods constituted a risk factor for COVID-19 infection and hospitalisation among HCWs in Sweden, and if so, whether such exposure amplified COVID-19 risk among immigrant HCWs. Addressing this question will help in better understanding of the factors underpinning COVID-19 inequalities among HCWs.

## Materials and methods

### Study design and population

**Figure 1:**
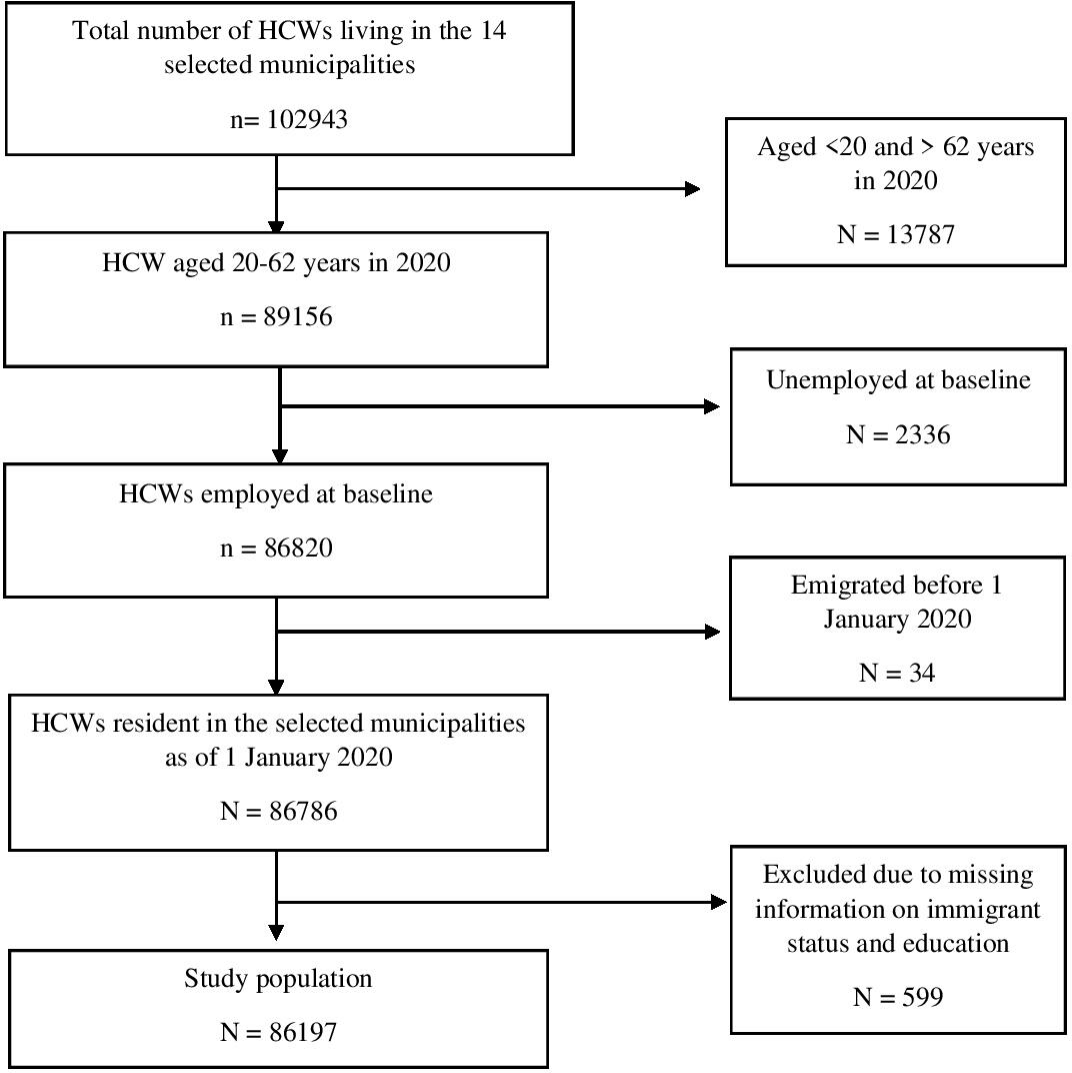
Flow chart showing the selection of the study population comprising healthcare workers (HCWs) aged 20–62 years and living in 14 selected Swedish municipalities as of 1 January 2020.

This longitudinal study was based on data from the SCIFI-PEARL project (Swedish COVID-19 Investigation for Future Insights—a Population Epidemiology Approach using Register Linkage). The SCIFI-PEARL project, described in detail elsewhere,^24^ covers the entire Swedish population and includes a unique linkage of regularly updated health and administrative data from multiple Swedish registers. We focused on data from HCWs who were living in 14 Swedish municipalities on 1 January 2020. The municipalities were Stockholm, Gothenburg, Malmö, Nacka, Lidingö, Solna, Sundbyberg, Järfälla, Danderyd, Sollentuna, Täby, Mölndal, Partille and Öckerö. The first three are the central areas of the three largest cities in Sweden. They have the highest total populations and proportions of immigrant residents, as well as the most pronounced residential segregation between immigrant and non-immigrant residents.^25^ The remaining municipalities, which are densely populated suburbs of Stockholm and Gothenburg, were selected because they too have a high proportion of immigrant residents. The study population was identified using municipality of residence from the Total Population Register (TPR), combined with four-digit Swedish Standard Occupational Classification (SSYK2012) codes for HCWs registered in the Longitudinal Integrated Database for Health Insurance and Labour Market Studies (LISA). Our sample was restricted to 86 187 individuals aged 20–62 years (in 2020) and employed as HCWs according to the most recent prepandemic (2019) employment information from LISA (figure 1). The HCWs consisted of all essential HCWs as defined by Billingsley *et al*,^26^ plus a few additions informed by previous studies^12 27^ (online supplemental table 1).

### Immigrant status and neighbourhood immigrant density

Country of birth for HCWs and their parents was obtained from the TPR and used to classify HCWs as non-immigrants or immigrants (ie, first-generation and second-generation immigrants). The non-immigrant group comprised individuals born to two Swedish-born parents, while the immigrant group referred to individuals with at least one parent not born in Sweden. This definition was chosen as research indicates that both first-generation and second-generation immigrants have a higher risk of COVID-19 than native-born individuals.^28^ Data from Statistics Sweden’s demographic statistical areas (DeSOs) were used to classify the residential area. DeSOs subdivide Sweden into 5984 geographical areas, each with between 700 and 2700 inhabitants. The subdivision is generally used to monitor segregation and socioeconomic conditions in small areas.^29^ There were a total of 1395 DeSOs in the 14 included municipalities. Statistics Sweden provided us with the DeSO in which each individual lived at the end of 2019. This information was linked to the cohort using the unique personal identification number. We calculated neighbourhood immigrant density as the proportion of immigrant residents in each DeSO and divided the HCWs into those living in immigrant-dominated (≥60% immigrants) neighbourhoods, mixed (40%–59% immigrants) or Swedish-dominated (<40% immigrants) neighbourhoods. This grouping is similar to that used in earlier research.^30^ We used the same definition of immigrants at both the individual and neighbourhood levels.

### COVID-19 outcomes

Our measurements of COVID-19 infection and hospitalisation relate to the first registered event per individual. We defined COVID-19 infection as having a positive test for SARS-CoV-2 in the national database of notifiable diseases (SmiNet) or a specialist healthcare encounter (visit or hospitalisation) with an International Classification of Diseases 10th revision, Swedish version code U07.1 or U07.2 in the National Patient Register (NPR) or the same code as an underlying or contributing cause of death in the Cause-of-Death Register. The event date was the earliest of these. COVID-19 hospitalisation referred to hospital admissions based on a primary or secondary diagnosis of COVID-19, with the event date being the date of hospital admission. Follow-up for each outcome extended from 1 January 2020 to the earliest of the outcome, emigration, death or 30 September 2022.

### Covariates

Covariates included demographic, socioeconomic and health characteristics of the study population. Demographic characteristics were from the TPR and included age (20–34, 35–44, 45–54, 55–62 years), sex (men, women) and municipality of residence (Stockholm plus 8 suburbs, Gothenburg plus 3 suburbs and Malmö). Socioeconomic characteristics included marital status (married/partnered, single or divorced/widowed/widower), highest education attained (primary, secondary or tertiary), annual household disposable income (divided into tertiles), occupational role (health professionals (physicians and dentists), health associate professionals (nurses and allied HCWs), healthcare support staff (assistant nurses, home-based personal care workers and ambulance staff)) and household size (one, two, three, four, or five or more household members). These variables were all measured in 2019 and extracted from the LISA and Statistics Sweden apartment registers (for household size). Health status data were from the NPR and included pre-existing medical conditions such as hypertension, diabetes, stroke, obesity, asthma, chronic obstructive pulmonary disease, pneumonia and psychiatric conditions. Individuals with at least one of the listed conditions diagnosed in specialist care between 2015 and 2019 were defined as having pre-existing comorbidities.

### Statistical analysis

We used frequencies and percentages to describe baseline characteristics and χ^2^ tests to assess group differences. We used multilevel mixed-effects Weibull survival regression for the association analyses, with DeSO random effects to account for neighbourhood clustering. We first examined the crude effects of neighbourhood immigrant density on COVID-19 outcomes before fitting an adjusted regression model that included age, sex, municipality of residence, marital status, education, income, occupational role, comorbidities and immigrant status. We assessed whether neighbourhood immigrant density amplified the risk of COVID-19 among immigrant HCWs by including an interaction term between neighbourhood immigrant density and immigrant status in the adjusted model. We used the log-likelihood ratio test to compare models with and without interaction terms and subsequently investigated associations between COVID-19 outcomes and a six-category variable denoting groups formed by the interaction of immigrant status and neighbourhood immigrant density, with non-immigrant HCWs living in Swedish-dominated neighbourhoods as the reference category. We performed several sensitivity analyses, including redefining neighbourhood immigrant density using other cut-offs, analysing the data with Cox regression with cluster-robust SEs, and restricting the data to only the first COVID-19 wave (1 January 2020 to 31 August 2020) to see if our findings were sensitive to changes during the pandemic. Kaplan-Meier survival curves were plotted to estimate survival probabilities. Associations were expressed as HRs, with 95% CIs. All statistical analyses were conducted using Stata V.17 (StataCorp).

## Results

The study population’s mean age was 42 years (SD 11.6); the majority being women (79%). Proportions of immigrants and non-immigrants were nearly equal (53% vs 47%). Half of the HCWs lived in Swedish-dominated neighbourhoods, while nearly a quarter (24%) resided in immigrant-dominated neighbourhoods. Relative to HCWs living in Swedish-dominated neighbourhoods, HCWs residing in immigrant-dominated neighbourhoods were less likely to have a tertiary education (77% vs 39%) and to be in the high income tertile (45% vs 14%), and more likely to work as healthcare support staff (30% vs 78%) and live in households with five or more residents (13% vs 26%) (table 1). The prevalence of comorbidities was slightly lower in HCWs living in Swedish-dominated neighbourhoods than in those residing in immigrant-dominated neighbourhoods (12% vs 16%).

**Table 1:** Baseline sociodemographic and socioeconomic characteristics and health status of healthcare workers (HCWs) aged 20–62 years on 1 January 2020 in Sweden, overall and by neighbourhood immigrant density grouping

	Total	Neighbourhood immigrant density		
	N=86 187	Swedish-dominated neighbourhoodsn=43 153	Mixed neighbourhoodsn=22 078	Immigrant-dominated neighbourhoodsn=20 956
	n (%)	%	%	%
Immigrant status				
Non-immigrants	40 628 (47.1)	63.0	44.6	17.2
Immigrants	45 559 (52.9)	37.0	55.4	82.8
Age (years)				
20–34	27 925 (32.4)	28.6	34.8	37.6
25–44	21 590 (25.1)	25.4	24.1	25.5
45–54	20 364 (23.6)	24.4	22.8	23.0
55–62	16 308 (18.9)	21.6	18.4	14.0
Sex				
Men	18 257 (21.2)	21.9	20.4	20.6
Women	67 930 (78.8)	78.1	79.6	79.4
Municipality of residence				
Stockholm	47 255 (54.8)	55.4	52.9	55.8
Gothenburg	25 817 (30.0)	37.0	22.3	23.4
Malmö	13 115 (15.2)	7.6	24.8	20.8
Marital status				
Married/cohabiting	34 462 (40.0)	42.9	36.7	37.5
Single	37 010 (42.9)	44.6	45.7	36.6
Divorced/widowed/widower	14 715 (17.1)	12.5	17.7	25.9
Highest education				
Primary	3778 (4.4)	1.9	4.0	9.9
Secondary	27 518 (31.9)	21.1	34.3	51.6
Tertiary	54 891 (63.7)	77.0	61.7	38.5
Annual household disposable income (tertiles)				
Low	28 558 (33.1)	22.2	34.5	54.2
Middle	28 775 (33.4)	32.5	36.4	31.8
High	28 854 (33.5)	45.3	29.0	13.9
Household size				
One	15 319 (17.8)	18.2	20.1	14.3
Two	22 438 (26.0)	27.2	28.4	21.2
Three	17 043 (19.8)	19.6	19.9	20.1
Four	17 511 (20.3)	22.0	18.8	18.4
Five or more	13 876 (16.1)	13.0	12.9	26.0
Pre-existing medical condition*				
No	74 558 (86.5)	87.8	86.2	84.3
Yes	11 629 (13.5)	12.2	13.8	15.7
Occupational roles†				
Health professionals	16 409 (19.0)	27.7	15.0	5.4
Health associate professionals	29 500 (34.2)	42.2	35.1	16.9
Healthcare support staff	40 278 (46.7)	30.1	49.9	77.7

* Pre-existing comorbidities include hypertension, diabetes, stroke, obesity, asthma, chronic obstructive pulmonary disease, pneumonia and psychiatric conditions.

† Health professionals include physicians and dentists; health associate professionals include nurses and allied HCWs (ie, chiropractors, naprapaths, occupational therapists, and health professionals not elsewhere classified), while healthcare support staff include assistant nurses, homebased personal care workers and ambulance staff.

Between 1 January 2020 and 30 September 2022, we recorded 39 746 COVID-19 infections and 860 hospitalisations. 99% of the COVID-19 infections were identified based on positive test results from SmiNet. Residing in immigrant-dominated or mixed neighbourhoods was not associated with an increased risk of COVID-19 infection, but living in these areas was associated with an elevated risk of COVID-19 hospitalisation (table 2). After controlling for potential confounders, the HRs for COVID-19 hospitalisation were 33% and 20% higher in HCWs who lived in immigrant-dominated and mixed neighbourhoods, respectively, compared with HCWs who lived in Swedish-dominated neighbourhoods (table 2). Immigrant HCWs also had a lower risk of COVID-19 infection, but a higher risk of COVID-19 hospitalisation than non-immigrant HCWs (table 3).

**Table 2:** Unadjusted and adjusted associations between neighbourhood immigrant density and COVID-19 infection and hospitalisation among healthcare workers in Sweden aged 20–62 years on 1 January 2020

Mixed-effects Weibull PH regression	No. of events	Person-years	COVID-19 infection		No. of events	Person-years	COVID-19-related hospitalisation	
			Unadjusted model	Adjusted model			Unadjusted model	Adjusted model*
			HR (95% CI)	HR (95% CI)			HR (95% CI)	HR (95% CI)
	N=39 746				N=860			
Neighbourhood immigrant densities								
Swedish-dominated neighbourhoods	20 090	93 900.3	Ref	Ref	336	117 289.5	Ref	Ref
Mixed neighbourhoods	10 368	47 700.8	1.02 (0.99 to 1.05)	1.02 (0.99 to 1.05)	229	59 913.9	1.34 (1.12 to 1.59)	1.20 (1.01 to 1.43)
Immigrant-dominated neighbourhoods	9288	45 352.2	0.97 (0.94 to 1.00)	0.98 (0.94 to 1.01)	295	56 709.2	1.80 (1.53 to 2.13)	1.33 (1.10 to 1.60)

Follow-up was from 1 January 2020 to 30 September 2022.

* Model adjusted for age, sex, municipality of residence, marital status, highest education, income, occupational role, household size, pre-existing medical conditions and immigrant status.

PH, proportional hazards.

**Table 3:** Unadjusted and adjusted associations between immigrant status and COVID-19 infection and hospitalisation among healthcare workers in Sweden aged 20–62 years on 1 January 2020

Mixed-effects Weibull PH regression	No. of events	Person-years	COVID-19 infection		No. of events	Person-years	COVID-19-related hospitalisation	
			Unadjusted model	Adjusted model*			Unadjusted model	Adjusted model*
			HR (95% CI)	HR (95% CI)			HR (95% CI)	HR (95% CI)
	N=39 746				N=860			
Immigrant status								
Non-immigrants	19 373	88 457.7	Ref	Ref	246	110 791.8	Ref	Ref
Immigrants	20 373	98 495.5	0.96 (0.94 to 0.98)	0.96 (0.94 to 0.98)	614	123 120.8	2.22 (1.91 to 2.57)	1.85 (1.57 to 2.18)

Follow-up was from 1 January 2020 to 30 September 2022.

* Model adjusted for age, sex, municipality of residence, marital status, highest education, income, occupational role, household size, pre-existing medical conditions and neighbourhood immigrant density.

PH, Proportional hazards.

In the analysis cross-classifying immigrant status with neighbourhood immigrant density, HCWs in most neighbourhood types had no increased risk of COVID-19 infection, except for non-immigrant HCWs living in immigrant-dominated neighbourhoods, who had a 6% higher risk of COVID-19 infection than non-immigrant HCWs living in Swedish-dominated neighbourhoods (table 4). For COVID-19 hospitalisation, there was around a 2-fold higher risk among immigrant HCWs regardless of the neighbourhood they lived in, compared with the reference group (table 4). There was no statistically significant increased risk among non-immigrant HCWs living in other neighbourhoods than Swedish-dominated neighbourhoods.

**Table 4:** Unadjusted and adjusted associations between intersectional strata cross-classing immigrant status and neighbourhood immigrant density and COVID-19 infection and hospitalisation among healthcare workers in Sweden aged 20–62 years on 1 January 2020

Mixed-effects Weibull PH regression	No. of eventsN=39 746	Person-years	COVID-19 infection		No. of eventsN=860	Person-years	COVID-19-related hospitalisation	
			Unadjusted model	Adjusted model*			Unadjusted model	Adjusted model*
			HR (95% CI)	HR (95% CI)			HR (95% CI)	HR (95% CI)
Immigrant status/neighbourhood immigrant density intersection†								
Non-immigrants/Swedish-dominated neighbourhood	12 893	59 226.6	Ref	Ref	160	74 123.4	Ref	Ref
Non-immigrants/mixed neighbourhood	4712	21 443.3	1.01 (0.97 to 1.05)	1.01 (0.98 to 1.05)	64	26 819.3	1.11 (0.83 to 1.49)	1.15 (0.86 to 1.55)
Non-immigrants/immigrant-dominated neighbourhood	1768	7787.8	1.06 (1.01 to 1.12)	1.06 (1.01 to 1.12)	22	9849.1	1.03 (0.66 to 1.62)	1.04 (0.66 to 1.63)
Immigrants/Swedish-dominated neighbourhood	7197	34 673.7	0.97 (0.94 to 1.00)	0.97 (0.94 to 0.99)	176	43 166.1	1.88 (1.52 to 2.33)	1.74 (1.40 to 2.16)
Immigrants/mixed neighbourhood	5656	26 257.5	1.00 (0.96 to 1.03)	0.99 (0.96 to 1.03)	165	33 094.7	2.30 (1.85 to 2.87)	2.15 (1.71 to 2.71)
Immigrants/immigrant-dominated neighbourhood	7520	37 564.3	0.93 (0.90 to 0.96)	0.93 (0.90 to 0.96)	273	46 860.1	2.68 (2.19 to 3.27)	2.43 (1.94 to 3.04)

Follow-up was from 1 January 2020 to 30 September 2022.

* Model adjusted for age, sex, municipality of residence, marital status, highest education, income, occupational role, household size and pre-existing medical conditions.

† The log-likelihood ratio test for the interaction between immigrant status and neighbourhood immigrant density was statistically significant in the model with COVID-19 infection as the outcome (p≤0.001), but not in the COVID-19-related hospitalisation model (p=0.475).

PH, Proportional hazards.

These findings remained largely consistent when we repeated the analysis using Cox regression with cluster-robust SEs and when using alternative neighbourhood immigrant density cut-offs (online supplemental tables 2–6). When the data were restricted to the first wave of the pandemic, an increased risk of COVID-19 infection was observed among HCWs living in immigrant-dominated neighbourhoods (adjusted HR 1.17; 95% CI 1.07 to 1.29) and the risk of hospitalisation was more pronounced (online supplemental table 7). In the six-category analysis, immigrant HCWs living in immigrant-dominated neighbourhoods were the only group more likely to have COVID-19 infection than the reference group of non-immigrant HCWs living in Swedish-dominated neighbourhoods (adjusted HR 1.28; 95% CI 1.16 to 1.42) (online supplemental table 8). The Kaplan-Meier plot for COVID-19 infection showed lower survival probabilities at the start of the pandemic among HCWs living in immigrant-dominated neighbourhoods than those living in Swedish-dominated neighbourhoods, with a switch occurring near the end of the second year (online supplemental figure 1).

## Discussion

Among HCWs in Sweden, we found that residing in immigrant-dominated neighbourhoods was not a risk factor for COVID-19 infection (except during the first wave of the pandemic), but that it was a risk factor for having COVID-19-related hospitalisation. When we examined whether living in immigrant-dominated neighbourhoods magnified the risk among immigrant HCWs, our data showed no indication of such an effect (except during the first wave of the pandemic). Instead, we found that regardless of the neighbourhood type, immigrant HCWs had a higher risk of COVID-19-related hospitalisation than non-immigrant HCWs, even after adjusting for age, sex, municipality of residence, marital status, education, household income, occupational role, household size and comorbidities.

During the early phase of the pandemic, reports of increased COVID-19 cases and deaths in communities dominated by immigrants and ethnic minorities flooded the literature.^19^,^2231^ Recent studies with longer follow-up show a shift in the trend. Spangler *et al*^32^ reported that the number of COVID-19 cases in communities dominated by black and Latin residents significantly decreased between the first and second wave of the pandemic. Bergmann *et al*^33^ found that the association between COVID-19 infection and the proportion of county residents who were foreign-born non-citizens in the USA was strongly positively related from April to July 2020, but was zero or slightly negative from August 2020 to February 2021. Another US-based study^34^ found that neighbourhoods with a higher percentage of black and Hispanic residents had higher COVID-19 incidence in wave 1, but not in wave 2. These findings are consistent with our result on the effect of neighbourhood immigrant density on COVID-19 infection. It could be that there was a depletion of susceptibility in immigrant-dominated neighbourhoods because of protection from recent infections in later waves, or that HCWs living in these areas took adequate precautions (eg, mask wearing and social distancing) to avoid infection, or that public health practices in these neighbourhoods improved as the pandemic progressed, such that living in these areas no longer posed an additional health risk to HCWs after the first wave. The increased infection risk observed during the first wave is most likely due to insufficient knowledge about the disease and its prevention,^35^ as well as delay in reacting and implementing preventive measures (eg, COVID-19 vaccination) both within and outside the work environment.^35^

In both the main result and when restricted to only the first wave, we found that HCWs living in immigrant-dominated neighbourhoods had a higher risk of COVID-19 hospitalisation than those living in Swedish-dominated neighbourhoods. This finding mirrors that from another Swedish-based study covering the period from February 2020 to October 2020, which found higher rates of COVID-19 hospitalisation in municipalities with high proportion of foreign-born residents.^23^ It seems, however, that the increased risk may be explained by individual-level factors rather than neighbourhood-level factors, as our data further showed an increased risk of COVID-19 hospitalisation in all immigrant HCWs regardless of where they lived. The argument that host-related factors such as age, sex, comorbidities and genetics are the primary determinants of severe COVID-19 has been put forth in the literature.^36^ Though we controlled for demographics and several important pre-existing comorbidities, it was not possible for us to account for the effect of genetics. Due to limitations of data availability, we were also unable to control for some other individual-level factors that have been linked to severe COVID-19, such as vitamin D deficiency^37^ and smoking.^38^ Future studies may need to explore the extent to which these factors contribute to increased risk of COVID-19 hospitalisation among immigrant HCWs in Sweden.

The risk of contracting an infection is thought to be especially high in immigrant-dominated neighbourhoods because of the high prevalence of unfavourable socioeconomic conditions (poverty, inadequate housing, limited access to health services, etc).^14 17^ Surprisingly, living in these neighbourhoods did not appear to increase the risk of COVID-19 infection among immigrant HCWs in our study. Instead, we found that only non-immigrant HCWs who were living in immigrant-dominated neighbourhoods were at a slightly higher risk of COVID-19 infection compared with non-immigrant HCWs who lived in Swedish-dominated neighbourhoods. This finding was not present in the first wave, implying that the change may have occurred later in the pandemic, probably after the second year of the pandemic, as evidenced in the result from the Kaplan-Meier survival curve. It is possible that as COVID-19 vaccination became widespread and the disease was no longer considered a major public health threat, this population group’s attitude towards the disease became more relaxed. Bergmann *et al*^33^ also noted that after COVID-19 vaccination became widely available, counties with higher proportions of white residents in the USA often reported a higher proportion of COVID-19 cases than counties with high proportions of black residents.

More than half (53%) of our study participants were of immigrant origin. This high figure is consistent with the proportion reported in another Swedish study^39^ and can be attributed to the definition of immigrants that we used (ie, both first and second generations), the very high immigrant rates in Sweden in recent decades, along with the focus on the more urban areas in Sweden and the restriction of the sample to essential HCWs.

We are not aware of other studies on neighbourhood immigrant density and COVID-19 outcomes that have specifically targeted HCWs. Therefore, our study makes a unique contribution to existing literature on the determinants of COVID-19 disparities among HCWs. Major strengths of the study include the use of large population-based register data, the use of a longitudinal design covering nearly four pandemic waves and the classification of neighbourhood types using DeSO, which is arguably a relevant operationalisation of neighbourhoods at small area level. We performed several sensitivity analyses, including using alternative model specifications, cut-offs for defining neighbourhood immigrant density and study periods. Our data, however, did not permit further disaggregation of the immigrant population (country of birth, first-generation vs second-generation immigrations), which would have provided a more nuanced understanding. Although we controlled for several important demographic and socioeconomic factors and comorbidities, we lacked data on some other individual-level characteristics (eg, smoking, vitamin D intake) that may underlie the remaining excess risk of COVID-19 hospitalisation that was observed among immigrant HCWs. We also did not account for the effect of COVID-19 vaccination in our study because we viewed it as a mediator; adjusting for it would block the pathway, and thus underestimate the effect of neighbourhood immigrant density and or being an immigrant on the outcomes. Although our study population comprised HCWs who were a priority group for COVID-19 testing throughout the pandemic, we cannot rule out the possibility that there may be differential access or testing propensities across the studied groups.^40^ It is worth noting that we did not account for a lockdown period in our analysis because mandatory lockdown was not enforced in Sweden during the pandemic.

In conclusion, our study showed no obviously increased risk of COVID-19 infection among HCWs living in immigrant-dominated neighbourhoods, but we found that living in these neighbourhoods constituted a clear risk for COVID-19-related hospitalisation. It seems that the increased risk has more to do with individual-level than neighbourhood-level factors, as the interaction analysis indicated an elevated risk of COVID-19-related hospitalisation in all immigrant HCWs regardless of where they lived. These findings highlight the need for more investigation to identify factors underpinning the excess risk of COVID-19 hospitalisation among immigrant HCWs in Sweden.

## Supplementary material

10.1136/bmjph-2024-001501 online supplemental figure 1

10.1136/bmjph-2024-001501 online supplemental table 1

## Data Availability

Data may be obtained from a third party and are not publicly available.
