# Supplementary material for: Neighbourhood immigrant density and COVID-19 infection and hospitalisation among healthcare workers in Sweden: a register-based observational study
Source: BMJ Public Health. 2025 Feb 26;3(1):e001501. doi: 10.1136/bmjph-2024-001501 (PMC11883869; doi:10.1136/bmjph-2024-001501)

Neighbourhood immigrant density and COVID-19 infection and hospitalisation among healthcare workers in Sweden - a registry-based observational study

Chioma Nwaru[1,2], Carl Bonander[1], Huiqi Li[1], Ailiana Santosa[1], Jesper Löve[1], Fredrik Nyberg[1]

Supplementary Figure 1: Kaplan-Meier Survival function for COVID-19 infection by neighbourhood immigrant density type

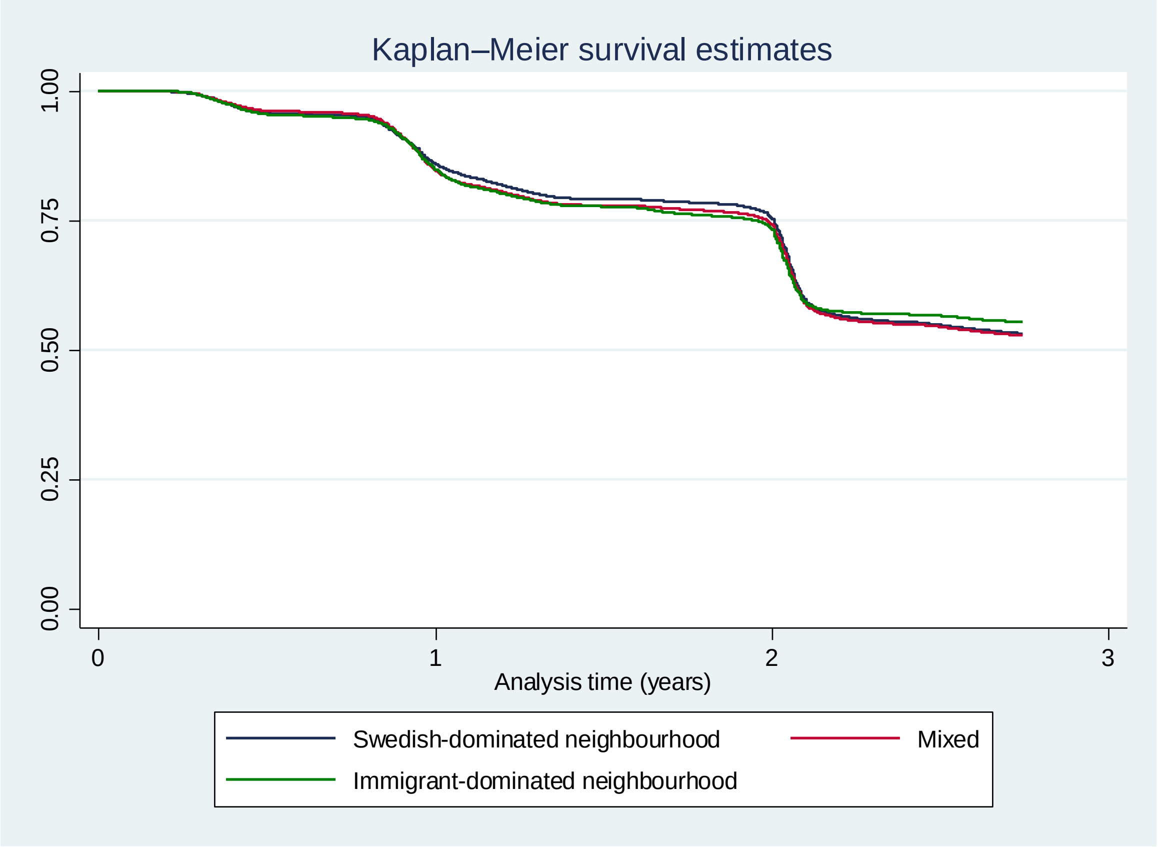

Supplement: online supplemental figure 1 [file bmjph-3-1-s001.pdf]
