# Supplementary material for: Neighbourhood immigrant density and COVID-19 infection and hospitalisation among healthcare workers in Sweden: a register-based observational study
Source: BMJ Public Health. 2025 Feb 26;3(1):e001501. doi: 10.1136/bmjph-2024-001501 (PMC11883869; doi:10.1136/bmjph-2024-001501)
Supplement: online supplemental table 1 [file bmjph-3-1-s002.pdf]

Neighbourhood immigrant density and COVID-19 infection and hospitalisation among healthcare workers in Sweden - a register-based observational study

Chioma Nwaru, Carl Bonander, Huiqi Li, Ailiana Santosa, Jesper Löve, Fredrik Nyberg

Supplementary Table 1: Essential healthcare occupations and codes based on the Swedish Standard for Classification of Occupations (SSYK 2012)

**SSYK12**	**Occupations**
2211	Specialist physicians
2212	Resident physicians
2213	General practitioners
2219	Other physicians
2260	Dentist
2221	Professional nurses
2222[a]	Professional midwives
2223	Anesthesia nurses
2224	District nurses
2226	Nurses- ambulance
2227	Nurses-geriatric
2228	Nurses- intensive care
2231	Nurses-operation
2232	Nurses-children
2235	Nurses-radiology
2239	Other specialist nurses
2289	Health professionals not elsewhere classified
2271	Chiropractors and naprapaths
2272	Physiotherapists
2273	Occupational therapist
2289	Health professionals not elsewhere classified

3250[a]	Dental hygienists
5350	Dental nurses
5321	Assistant nurses, homecare, and homes for the elderly
5322	Assistant nurses, rehabilitation
5323	Assistant nurses, hospital ward
5324	Assistant nurses, clinic
5325	Assistant nurses, children
5326	Ambulance attendants
5330	Home-based personal care and related workers

The selection of the occupations was based on the work of Billingsley et al.[1]

[a] The author's own additions based on previous research.[2]

Reference:

1. Billingsley S, Brandén M, Aradhya S, Drefahl S, Andersson G, Mussino E. Deaths in the frontline: Occupation-specific COVID-19 mortality risks in Sweden. Stockholm Reports in Demography 2020. https://doi.org/10.17045/sthlmuni.12816065.v2.
2. Mutambudzi M, Niedwiedz C, Macdonald EB, et al. Occupation and risk of severe COVID-19: a prospective cohort study of 120 075 UK Biobank participants. Occup Environ Med 2021;78:307-314.

Supplementary Table 2: Unadjusted and adjusted associations between neighbourhood immigrant densities and COVID-19 infection and hospitalisation among healthcare workers in Sweden aged 20 to 62 years on 1 January 2020. Follow-up was from 1 January 2020 to 30 September 2022. Result obtained with standard Cox regression with vce (cluster var) specification.

	COVID-19 infection		COVID-19-related hospitalisation	
	Unadjusted model	Adjusted model*	Unadjusted model	Adjusted model*
	HR (95%CI)	HR (95%CI)	HR (95%CI)	HR (95%CI)
Neighbourhood immigrant density				
Swedish-dominated neighbourhoods	Ref	Ref	Ref	Ref
Mixed neighbourhoods	1.02 (0.99 – 1.05)	1.02 (1.00 – 1.05)	1.33 (1.12 – 1.58)	1.20 (1.00 – 1.43)
Immigrant-dominated neighbourhoods	0.96 (0.93 – 1.00)	0.98 (0.95 – 1.02)	1.81 (1.54 – 2.14)	1.33 (1.09 – 1.61)

* Model adjusted for age, sex, municipality of residence, marital status, highest education, income, occupational role, household size, pre-existing medical conditions, and immigrant status.

Supplementary Table 3: Unadjusted and adjusted associations between immigrant status and COVID-19 infection and hospitalisation among healthcare workers in Sweden aged 20 to 62 years on 1 January 2020. Follow-up was from 1 January 2020 to 30 September 2022. Result obtained with standard Cox regression with vce (cluster var) specification.

	COVID-19 infection		COVID-19-related hospitalisation	
	Unadjusted model	Adjusted model*	Unadjusted model	Adjusted model*
	HR (95%CI)	HR (95%CI)	HR (95%CI)	HR (95%CI)
Immigrant status				
Non-immigrants	Ref	Ref	Ref	Ref
Immigrants	0.95 (0.93 – 0.97)	0.96 (0.94 – 0.98)	2.24 (1.93 – 2.60)	1.85 (1.58 – 2.17)

* Model adjusted for age, sex, municipality of residence, marital status, highest education, income, occupational role, household size, pre-existing medical conditions, and neighbourhood immigrant density.

Supplementary Table 4: Unadjusted and adjusted associations between immigrant status/neighbourhood immigrant intersection and COVID-19 infection and hospitalisation among healthcare workers in Sweden aged 20 to 62 years. Follow-up was from 1 January 2020 to 30 September 2022. Result obtained with standard Cox regression with vce (cluster var) specification.

	COVID-19 infection		COVID-19-related hospitalisation	
	Unadjusted model	Adjusted model*	Unadjusted model	Adjusted model*
	HR (95%CI)	HR (95%CI)	HR (95%CI)	HR (95%CI)
Immigrant status/neighbourhood immigrant density interaction				
Non-immigrants/ Swedish-dominated neighbourhood	Ref	Ref	Ref	Ref
Non-immigrants /mixed neighbourhood	1.01 (0.97 – 1.04)	1.01 (0.97 – 1.05)	1.10 (0.82 – 1.48)	1.15 (0.85 – 1.55)
Non-immigrants /immigrant-dominated neighbourhood	1.05 (1.00 – 1.10)	1.07 (1.02 – 1.13)	1.03 (0.67 – 1.59)	1.04 (0.67 – 1.61)
Immigrants /Swedish-dominated neighbourhood	0.95 (0.93 – 0.98)	0.97 (0.95 – 1.01)	1.88 (1.53 – 2.32)	1.74 (1.41 – 2.14)
Immigrants/mixed neighbourhood	1.00 (0.96 – 1.03)	1.00 (0.97 – 1.04)	2.30 (1.85 – 2.86)	2.15 (1.70 – 2.70)
Immigrants /immigrant-dominated neighbourhood	0.93 (0.89 – 0.97)	0.92 (0.89 – 0.97)	2.69 (2.20 – 3.29)	2.42 (1.93 – 3.05)

* Model adjusted for age, sex, municipality of residence, marital status, highest education, income, occupational role, household size, and pre-existing medical conditions

Supplementary Table 5: Unadjusted and adjusted associations between neighbourhood immigrant density and COVID-19 infection and hospitalisation among healthcare workers in Sweden aged 20 to 62 years on 1 January 2020. Follow-up was from 1 January 2020 to 30 September 2022. Results from Mixed-effects Weibull survival regression.

	COVID-19 infection		COVID-19-related hospitalisation	
	Unadjusted model	Adjusted model*	Unadjusted model	Adjusted model*
	HR (95%CI)	HR (95%CI)	HR (95%CI)	HR (95%CI)
Low immigrant-density neighbourhood (≤ 65% immigrants)	Ref	Ref	Ref	Ref
High immigrant-density neighbourhood (> 65% immigrants)	0.95 (0.92 – 0.98)	0.95 (0.92 – 0.98)	1.59 (1.36 – 1.87)	1.16 (0.98 – 1.37)
Low immigrant-density neighbourhood (< 80% immigrants)	Ref	Ref	Ref	Ref
High immigrant-density neighbourhood (80+% immigrants)	0.92 (0.88 – 0.96)	0.91 (0.87 – 0.95)	1.54 (1.26 – 1.88)	1.10 (0.90 – 1.35)

* Model adjusted for age, sex, municipality of residence, marital status, highest education, income, occupational role, household size, pre-existing medical conditions, and immigrant status.

Supplementary Table 6: Unadjusted and adjusted associations between immigrant status/neighbourhood immigrant intersection and COVID-19 infection and hospitalisation among healthcare workers in Sweden aged 20 to 62 years on 1 January 2020. Follow-up was from 1 January 2020 to 30 September 2022. Results from Mixed-effects Weibull survival regression.

	COVID-19 infection		COVID-19-related hospitalisation	
	Unadjusted model	Adjusted model*	Unadjusted model	Adjusted model*
	HR (95%CI)	HR (95%CI)	HR (95%CI)	HR (95%CI)
Immigrant status/neighbourhood immigrant density interaction				
Non-immigrant/ low immigrant-density (≤ 65%) neighbourhood	Ref	Ref	Ref	Ref
Non-immigrant /high immigrant-density (> 65%) neighbourhood	1.01 (0.95 – 1.08)	1.05 (0.99 – 1.12)	1.06 (0.63 – 1.76)	0.98 (0.59 – 1.64)
Immigrant/low immigrant-density (≤ 65%) neighbourhood	0.97 (0.95 – 0.99)	0.98 (0.95 – 1.00)	2.07 (1.76 – 2.44)	1.88 (1.59 – 2.23)
Immigrant /high immigrant-density (> 65%) neighbourhood	0.92 (0.89 – 0.96)	0.90 (0.87 – 0.94)	2.57 (2.13 – 3.11)	2.22 (1.80 – 2.75)
Non-immigrant / low immigrant-density (<80%) neighbourhood	Ref	Ref	Ref	Ref
Non-immigrant /high immigrant-density (80+ %) neighbourhood	1.00 (0.90 – 1.11)	1.07 (0.95 – 1.16)	1.23 (0.54 – 2.78)	1.13 (0.50 – 2.55)
Immigrant /low immigrant-density (< 80%) neighbourhood	0.97 (0.95 – 0.99)	0.97 (0.95 – 0.99)	2.17 (1.85 – 2.53)	1.95 (1.65 – 2.29)
Immigrant /high immigrant-density (80+%) neighbourhood	0.89 (0.85 – 0.93)	0.87 (0.83 – 0.90)	2.51 (2.00 – 3.14)	2.15 (1.69 – 2.73)

* Model adjusted for age, sex, municipality of residence, marital status, highest education, income, occupational role, household size, and pre-existing medical conditions

Supplementary Table 7: Unadjusted and adjusted associations between neighbourhood immigrant density and COVID-19 infection and hospitalisation during the first wave of the pandemic (1 January 2020 to 31 August 2020) among healthcare workers in Sweden aged 20 to 62 years on 1 January 2020. Results from Mixed-effects Weibull survival regression.

	No. of events N = 3,888	Person-years	COVID-19 infection		No. of events N = 342	Person-years	COVID-19-related hospitalisation	
			Unadjusted model	Adjusted model*			Unadjusted model	Adjusted model*
			HR (95%CI)	HR (95%CI)			HR (95%CI)	HR (95%CI)
Neighbourhood immigrant density								
Swedish-dominated neighbourhoods	1956	28117.9	Ref	Ref	127	28631.6	Ref	Ref
Mixed neighbourhoods	907	14416.2	0.90 (0.83 – 0.99)	1.01 (0.93 – 1.10)	82	14646.5	1.26 (0.95- 1.69)	1.24 (0.92 – 1.66)
Immigrant-dominated neighbourhoods	1025	13641.8	1.08 (0.99 – 1.17)	1.17 (1.07 – 1.29)	133	13881.3	2.15 (1.66 – 2.77)	1.77 (1.32 – 2.38)

* Model adjusted for age, sex, municipality of residence, marital status, highest education, income, occupational role, household size, pre-existing medical conditions, and immigrant status.

Supplementary Table 8: Unadjusted and adjusted associations between immigrant status/neighbourhood immigrant intersection and Covid-19 infection and hospitalisation during the first wave of the pandemic (1 January 2020 to 31 August 2020) among healthcare workers in Sweden aged 20 to 62 years on 1 January 2020. Results from Mixed-effects Weibull survival regression.

	No. of events N = 3,888	Person-years	COVID-19 infection		No. of events N = 342	Person-years	COVID-19-related hospitalisation	
			Unadjusted model	Adjusted model*			Unadjusted model	Adjusted model*
			HR (95%CI)	HR (95%CI)			HR (95%CI)	HR (95%CI)
Immigrant status/neighbourhood immigrant density interaction								
Non-immigrant/ Swedish-dominated neighbourhood	1218	17723.5	Ref	Ref	66	18047.9	Ref	Ref
Non-immigrant /mixed neighbourhood	395	6428.0	0.90 (0.80 – 1.01)	1.04 (0.92 – 1.16)	25	6530.9	1.05 (0.66 – 1.68)	1.19 (0.74 – 1.90)
Non-immigrant/ immigrant-dominated neighbourhood	135	2366.6	0.83 (0.69 – 1.00)	0.98 (0.81 – 1.17)	7	2397.9	0.80 (0.37 – 1.75)	0.91 (0.41 – 2.00)
Immigrant /Swedish-dominated neighbourhood	738	10394.4	1.03 (0.94 – 1.13)	1.04 (0.95 – 1.14)	61	10583.7	1.57 (1.11 – 2.23)	1.46 (1.03 – 2.08)
Immigrant /mixed neighbourhood	512	7988.2	0.93 (0.83 – 1.04)	1.05 (0.94 – 1.17)	57	8115.6	1.92 (1.34 – 2.75)	1.93 (1.33 – 2.81)
Immigrant / immigrant-dominated neighbourhood	890	11275.2	1.14 (1.04 – 1.26)	1.28 (1.16 – 1.42)	126	11483.4	2.98 (2.19 – 4.04)	2.96 (2.09 – 4.19)

* Model adjusted for age, sex, municipality of residence, marital status, highest education, income, occupational role, household size, and pre-existing medical conditions.
